# Simulation of Age‐Related Limitations of Patients in Patient‐Centred Dental Education

**DOI:** 10.1111/eje.13075

**Published:** 2025-02-17

**Authors:** Ina Nitschke, Martin Holter, Bernhard Sobotta, Julia Jockusch

**Affiliations:** ^1^ Gerodontology Section, Department of Prosthodontics and Materials Science Leipzig University Leipzig Germany; ^2^ Center for Gerontology & Healthy Longevity Center University of Zurich Zurich Switzerland

**Keywords:** age experience, age simulation, ageism, geriatric dentistry, gerodontolgy, understanding for seniors

## Abstract

**Introduction:**

Young people, including dental students, generally have little experience with older people who suffer from multimorbidity with age‐related functional and/or cognitive limitations. For this reason, the ‘Gero‐Parcours’ (GP) as an educational instrument of multi‐staging simulation teaching was developed for students to experience ageing and its limitation.

**Materials and Methods:**

The GP consists of different stations (e.g., hearing and visual impairments, teeth brushing by third‐party cleaners, emotion), which are completed within 15 minutes each by two students at the same time supervised by an educated professional. Students' assessment of the GP at the University of Leipzig, Germany between 2021 and 2024 was evaluated by using written questionnaires.

**Results:**

Student's experiences and perceptions of the GP highlighted its positive influence on their understanding of ageing. Most students stated that the course enriched their professional knowledge and dispelled misconceptions about ageing in patients. Some students expressed personal concerns about ageing. Students found the transfer from a wheelchair to a dental chair station most beneficial for their future work life, while the clinical nutrition station received the lowest rating.

**Discussion:**

The GP illustrates ageing with its limitations. The students and supervisors appreciated this practical training. However, when planning a GP, it is necessary to provide the necessary resources for the course. Students from the higher years can also be recruited and trained as supervisors.

**Conclusion:**

The GP can be easily adapted to the number of students and supervisors as well as to the time available. As the students found the GP to be a helpful educational instrument, the authors recommend including it as mandatory in the dental education curriculum.

## 1 Introduction

There is evidence in everyday life and in the literature that physicians would be reluctant to enter or seek employment in the specialty of geriatrics [1]. In a systematic review, it was shown that many factors influence the decision of students to engage with older people or patients in very different ways. Women seem to be more likely than men to do so, but financial aspects and the reputation of the subject seem to play a role, too [1]. Significantly more students were interested in geriatrics at a medical school with a geriatrics department than at a medical school without one [2].

Reports from other studies show that more students were willing to consider a career with older patients when they underwent a geriatric or general medicine clerkship [3, 4]. After an eight‐workday clinical training program in geriatric medicine, to which medical students were randomly assigned, a statistically significant increase in interest in the speciality of geriatrics was found [3, 4]. Similarly, two other studies reported an increase in interest after a 5‐week and 1‐month internship in geriatric nursing, respectively, although statistical significance was not mentioned [5, 6]. There was no difference in interest between students who completed a 4‐week geriatric rotation within an 8‐week primary care clerkship and those who did not [7].

In Germany, dental students perform dental treatments under supervision from the 7th to the 10th semester of their studies. Patients attending the student's clinic usually include older people who are healthy enough to visit the clinic and sufficiently patient to cooperate through extended treatment sessions. They usually include older people, although they are healthy enough. Occasionally an extra‐mural is offered whereby students attend to dental needs of residents of a long‐term care facility. However, the teaching time for these activities is rather limited.

The speciality of senior dentistry comprises the part of dentistry that deals specifically with the dental care of retired older people. In terms of prevention and treatment concepts, senior dentistry adapts to the continuous process of ageing and its functional and cognitive limitations. It involves collaboration among professionals from health, nutrition and nursing sciences, as well as geriatrics and medical ethics, working together in a multidisciplinary and interdisciplinary approach. This field addresses scientific inquiries related to oral and general health, as well as the quality of life of older adults and the very old. The terms ‘Gerostomatology’ or ‘Gerodontology’ can be used synonymously. In contrast, geriatric dentistry is a subfield of senior dentistry, the dentistry for dependent older adults [8].

Until the introduction of the new licensing regulations (2022), senior dentistry was not taught on a mandatory basis in Germany. Only four out of the 30 German dental schools had voluntary offerings, with a similar situation in Austria. In Switzerland, on the other hand, senior dentistry has been a mandatory training subject for many years, which is also examined in writing in the central Swiss state examination [9, 10]. Additionally, education in senior dentistry could have a positive impact on students' perceptions of age and the ageing process [11]. Different training concepts have been developed [12, 13, 14].

Most young students may have occasional contact with older people in their family, but these older relatives are more likely to be younger grandparents than high‐aged great‐grandparents. Consequently, there are only sporadic interactions with aged and very‐aged people. As a result, students often lack a comprehensive understanding of the challenges and difficulties associated with ageing and old age.

This absence of insight extends across various domains, encompassing not only medical, pharmacological, nursing and social fields but also product design, such as food packaging and automobile construction. This has prompted a growing interest in simulating the experiences and limitations of older individuals in recent decades.

Ageing suits which simulate different age groups requiring varying degrees of effort have emerged and, for example, automakers have more frequently used people in ageing suits in the development of new cars to enter and exit the vehicle [15]. The simulation of ageing has been shown to increase empathy for people who are ageing [15]. There is also the question of whether the age suit really comes close to old age. Different studies demonstrated that ageing suits offer a realistic experience of the physical limitations associated with ageing. The learner's exposure to and occupation with the suit alone created attention for the subject and initiated a learning experience.

Computer‐based learning modules [16, 17] or real patient situations with medically oriented actors [18] are also used to increase understanding of the elderly. Simulation games help students reduce their reservations and show empathy and a positive attitude towards elderly patients [17].

The basis of this study is a multi‐staging simulation teaching in which the participants have to work through a structured path with various stations and tasks, which is called ‘Gero‐Parcours’ (GP).

The term ‘parcours’, derived from the French word for ‘course’ or ‘route’, is often used to describe a sequence of activities or exercises designed to challenge and develop specific skills. In the context of our study, the term ‘parcours’ refers to a structured series of tasks or experiences that participants go through to gain impressions and experiences of age‐related limitations.

The aim of the present study was (a) to report on the dental students' assessment of the ‘Gero‐Parcours’ (GP), a multi‐staging simulation teaching, they underwent shortly before starting their dental clinical training as part of their training in senior dentistry and (b) to evaluate supervisors' feedback on the GP to assure internal quality of the GP.

## 2 Materials and Methods

### 2.1 Study Setting

In Germany, dental education includes clinical treatment of patients starting in the 7th semester. At the University of Leipzig, Germany, this clinical phase is preceded by a 4‐hour age awareness simulation training, known as the ‘Gero‐Parcours’ (GP), aimed at fostering understanding and empathy towards the ageing process among students.

The GP as an educational instrument consists of 22 topics within five domains, described in detail in Tables S1–S5.

An ‘educational instrument’ is a teaching tool or pedagogical resource used in educational processes to support and enhance learning. This includes interactive simulations as the GP, which are designed to assist educators in delivering instructional content and to help learners understand and internalise the material.

Between 2021 and 2024, dental students at the University of Leipzig, Germany participated in training circuits consisting of 11 stations, supervised by 12 staff members. From a pool of 22 potential topics (Tables S1–S5), 15 topics were selected and tailored to the students' interests, learning objectives and available resources. Multiple topics could be grouped into a single station based on their content, allowing for efficient coverage of the material. This flexible structure enables the main supervisor to customise the selection and grouping of stations each year, depending on the available time (4 h at the University of Leipzig, Germany) and staff. Two students can be supported per station.

### 2.2 Survey of Dental Students

In the present study, dental students' assessments after completion of the GP were evaluated from academic years (2021, 2022, 2023, and 2024). The requirements for students and the setting of the GP were the same in all four years covered by the study. A structured written questionnaire with open and closed questions was given to the students after the completion of the last station of the GP each year.

The questionnaires included 27 closed and 3 open questions. The closed questions aimed not only at evaluating individual topics but also at assessing the usefulness of the topic areas for their own work. Additionally, the questionnaires captured evaluations of the supervisors, the equipment and the organisation of the GP. In the open questions, students could share their impressions, suggestions and criticisms of the GP or the supervisors.

### 2.3 Survey of Supervisors

Students who completed the GP were accompanied by individually trained supervisors during their participation in each of the stations. Supervisors can be dentists, dental assistants, dental hygienists or higher‐semester students. The supervisors were trained for their tasks at the stations. The better their understanding of the learning objective at their stations is, the better they will be able to supervise the students.

Table 3 provides an overview of the closed questions from the questionnaire for supervisors. Additionally, an open question regarding the supervisors' suggestions for improving the GP was asked (Table 4). Finally, the supervisors assessed the students' interest in the GP (Table 5).

The effectiveness of the supervisors was ensured, evaluated and further developed through various measures in the run‐up to, during and after the GP.

In the run‐up to the GP, the supervisors were prepared for their task by defining the goals and criteria of the GP that were to be achieved through the use of supervisors. In addition, the supervisors were deployed in areas in which they had the necessary qualifications and experience or for which they were prepared through additional internal training. During the unit, ongoing observation of the supervisors during the GP and direct feedback by the responsible professor ensured that the quality of the support was maintained. Observations and recordings of interactions between students and supervisors as well as post‐event feedback sessions with supervisors were introduced to discuss the experience and adjust where necessary. Furthermore, an evaluation form was developed to collect participant feedback. After the GP, the evaluated results, experiences and feedback were reflected upon. The accompanying evaluation, which was analysed in this paper, also made it possible to provide targeted feedback to the supervisors and to continuously adapt the GP.

Additionally, the supervisors were asked about their impressions (for the first year 2021 only) with a structured written survey with open and closed questions.

### 2.4 Statistical Considerations

Descriptive analyses (frequencies, means and standard deviations [SD], median and range) were used to describe the evaluation of dental students' assessment of the GP. Missing data (*n* = 1 for gender and age) were replaced with median age and female gender (predominantly female participants). Data were collected electronically and analysed using SPSS version 23 (IBM SPSS Statistics for Windows, Armonk, NY, USA) and Microsoft Excel.

### 2.5 Ethical Considerations

Participants (students and supervisors) were given the choice to take part in the research and were provided with comprehensive information regarding the purpose of the survey, emphasising voluntary participation. Measures were taken to ensure that there was no risk of coercion for students or staff to respond. The questionnaire data were collected anonymously, thereby preventing any possibility of tracing responses back to individual participants.

The evaluation of the teaching unit does not require an additional declaration of consent from the student but is a measure to ensure the quality of teaching. Therefore, no ethics vote of the responsible Ethics Committee (CEC) of the University Leipzig, Germany was necessary.

## 3 Results

### 3.1 Assessment of the Students

#### 3.1.1 General Information

Within the 4‐year evaluation period, 195 students (female *n* = 131, 67.2%, male *n* = 64, 32.8%; mean age ± SD: 24.0 ± 3.4 years, median age (range): 23.0 years (range 20–41 years); one missing data for gender and one for age were replaced by female and the median age) participated in the age awareness‐simulation training of the GP (Table 1).

**TABLE 1:** Overview of study participants in every evaluation year stratified by age and gender.

Evaluation year	Students [n]			Age (years)	
	Female	Male	Total	Mean ± SD	Median (range)
2021	26	22	48	24.2 ± 4.0	22 (21 – 41)
2022	34	13	47	23.9 ± 3.6	23 (21 – 38)
2023	38	13	51	24.0 ± 3.3	23 (20 – 36)
2024	33	16	49	24.1 ± 2.6	23 (21 – 31)
Total	131	64	195	24.0 ± 3.4	23 (20 – 41)

Most of the students were in their seventh semester (*n*_total_ = 189, 96.9%) and two students in 2021, 2022, and 2023 each stated that they were in their ninth semester (*n*_total_ = 6, 3.1%). These students were repeating the seventh semester but indicated themselves as ninth‐semester students, which corresponds to their personal study period. In 2024 all students were in their seventh semester.

#### 3.1.2 Prior Knowledge of Dental Students About Older Persons

The students had different experiences in dealing with people with special care needs. In total 70 of the 195 students (35.9%) (2021: *n* = 16, 33.3%, 2022: *n* = 12, 25.5%, 2023: *n* = 13, 25.5%, 2024: *n* = 29, 59.2%) had already provided care in a long‐term care facility. Ninety‐one of the 195 students (46.7%) (2021: *n* = 17, 35.4%, 2022: *n* = 24, 51.1%, 2023: *n* = 21, 41.2%, 2024: *n* = 29, 59.2%) had already helped an older person from a wheelchair to a chair. Only 49 students of the 195 students (25.1%) (2021: *n* = 6, 12.5%, 2022: *n* = 7, 14.9%, 2023: *n* = 16, 31.4%, 2024: *n* = 20, 40.8%) had brushed an older person's teeth (third‐party cleaning).

#### 3.1.3 Dental Students' Impressions About the GP

Students were asked open‐ended questions about their first impressions after the course. Their multiple responses to the question were grouped into seven categories and ranked according to their importance. Four students provided no information (Table 2).

**TABLE 2:** First impression of the “Gero‐Parcours” by dental students (*n* = 195) with examples of their responses to the question “How useful do you think the course is for understanding ageing.” grouped into seven categories and an indication of the frequency of mentioning.

Ranking	Grouped categories of first impressions	Examples of multiple responses by dental students	Year				Total
			2021	2022	2023	2024	
			(*n* = 48)	(*n* = 47)	(*n* = 51)	(n = 49)	(*n* = 195)
			(*n*/%)	(*n*/%)	(*n*/%)	(*n*/%)	(*n*/%)
#1	Professional knowledge enhancement	more understanding in professional matters I will need for my clinical future corrected misconceptions about aging in patients	19/39.6	24/51.1	16/31.4	5/10.2	64/32.8
#2	Personal concern about aging	experience of how difficult aging can be Perception of barriers to going to a doctor's office older people are not given enough respect	11/22.9	11/23.4	12/23.5	17/34.7	51/26.2
#3	Course appraisal	instructive was fun informative well organized	11/22.9	8/17.0	12/23.5	18/36.7	49/25.1
#4	Generally positive ratings	interesting insight very useful I was surprised	3/6.3	3/6.4	9/17.6	7/14.3	22/11.3
#5	No information provided		3/6.3	1/2.1	2/3.9	2/4.1	8/4.1
#6	Course Critique	did not help to understand older people better	1/2.1	0	0/0	0/0	1/0.5

**FIGURE 1:**
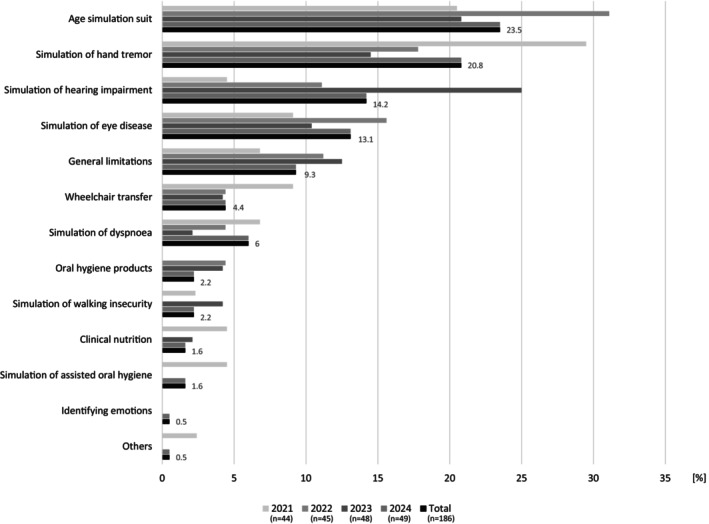
Students' assessment (*n*_total_ = 186) of the most impressive stations of the ‘Gero‐Parcours’ stratified by year (2021–2024) and in total. The ‘Gero‐Parcours’ stations simulation of hemiplegia, transfer aids, identifying emotions, gerostomatological aids and management of prosthesis have not been mentioned in the whole observation period.

Students' assessment of the most impressive GP stations revealed that the station with the age simulation suit AgeMan (Age Suit Germany GmbH, Saarbrücken, Germany) was perceived in total by 23.5% (2021: 20.5%, 2022: 31.1%, 2023: 20.8%, 2024: 21.7%) of the students as the most impressive, followed by the station with the hand tremor simulation (total: 20.8%, 2021: 29.5%, 2022: 17.8%, 2023: 14.5%, 2024: 21.7%) (Figure 1).

**FIGURE 2:**
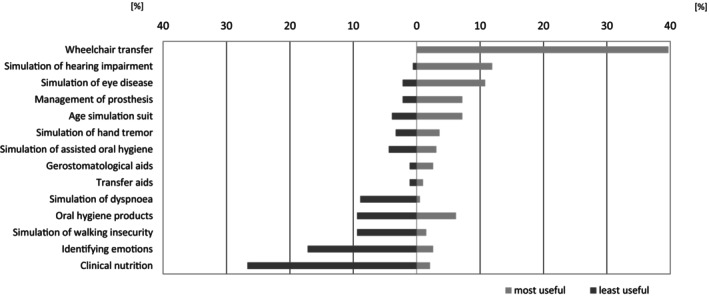
The ‘Gero‐Parcours’ stations considered most useful (*n* = 194) and least useful (*n* = 180) by the students (*n* = 195) for their future everyday professional life.

**FIGURE 3:**
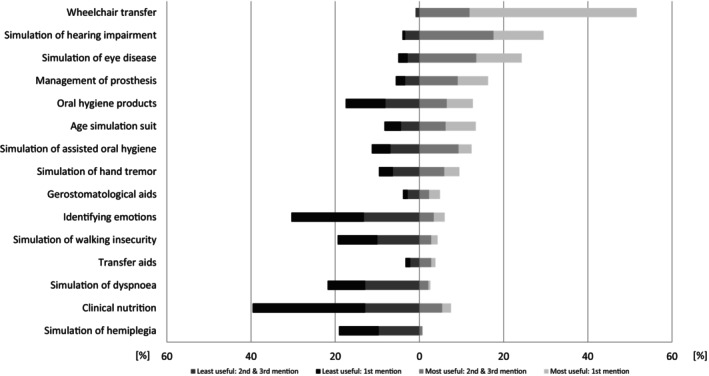
Students' (*n* = 195) assessment of the first most useful (*n* = 194), second most useful (*n* = 193) and third most useful (*n* = 193), and first least useful (*n* = 180), second most useful (*n* = 166) and third least useful (*n* = 153) stations of the ‘Gero‐Parcours’ for their future professional life.

The students were asked to name the three most useful and three least useful stations for their future professional life. The ranking is presented in Figures 2 and 3.

**FIGURE 4:**
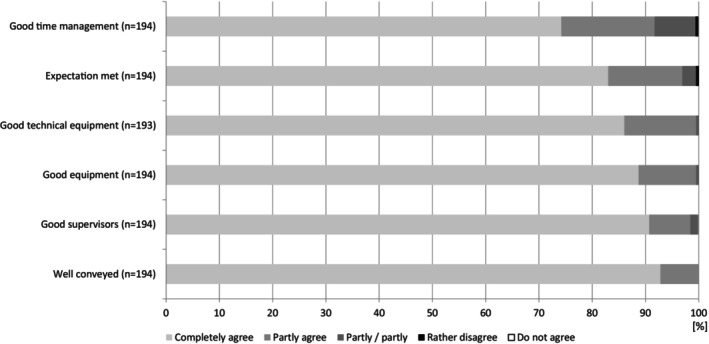
Evaluation of the teaching unit ‘Gero‐Parcours’ by the students (*n* = 195) for the complete evaluation period 2021–2024 per item in percent (%).

At the end of the survey, the students assessed the time management, the general facilities, technical equipment, the professional quality of the teachers and the teaching quality of the GP course. When asked if expectations for the course were met, most students fully agreed (*n* = 161, 83.0%) (2021: *n* = 38, 79.2%, 2022: *n* = 35, 74.5%, 2023: *n* = 48, 94.1%, 2024: *n* = 40, 81.6%) or agreed (*n* = 27, 13.9%) (2021: *n* = 6, 12.5%, 2022: *n* = 11, 23.4%, 2023: *n* = 1, 2.0%, 2024: *n* = 9, 18.4%) (Figure 4). In this study, 98.5% (*n* = 192) of the students would recommend the age awareness simulation training (2021: *n* = 46, 95.8%, 2022: *n* = 48, 100%, 2023: *n* = 51, 100%, 2024: *n* = 48, 98.0%).

Of the 195 students, 109 students (55.9%) gave feedback to the team of supervisors. In summary, 60 students (55.0%) praised the GP and 23 (21.1%) commented positively on the course design. Eight students (7.3%) gave suggestions on time management while one (0.9%) rated time management as positive, eight (7.3%) gave critical comments on the course design and nine students (8.3%) rated the supervisors positive.

### 3.2 Assessment of the Supervisors

Supervisors of the GP (*n* = 11) were assessed once in 2021. The choice of supervisors (female *n* = 8, 72.7%; mean age 31.8 ± 8.7 years, median age: 30 years (range 23–51 years)) was random and was based on the clinic schedule on the teaching days. It did not matter whether they were students, dentists or others. What was important was that sufficient expertise because of the supervisors' profession or training was available for the different stations. There were 12 supervisors (3 dentists, 4 students, 2 dental technicians, 1 dental nurse, 1 dental nurse in training and 1 clinical dietitian) on the individually selectable GP track with 11 stations, with one supervisor not participating in the survey.

The survey of the GP supervisors (*n* = 11) revealed that many were satisfied with their assignments at their stations or the available equipment, and felt comfortable with the work with the students. Most felt that they did not have enough time to prepare for their specific station and therefore did not feel well prepared at all (Table 3).

**TABLE 3:** Evaluation of the course ‘Gero‐Parcours’ by the supervisors.

	*n*	%
*Did you have enough time to prepare for your station?*		
Yes	1	9.1
No	10	90.9
*Did you feel well prepared for your station?*		
Yes	1	9.1
No	10	90.9
*Was the time (15 min) at your station adequate?*		
Yes	8	72.7
No, too short	2	18.2
*How satisfied were you with the equipment in your station?*		
Very satisfied	9	81.8
Rather satisfied	2	18.2
Rather not satisfied	0	0
Not satisfied	0	0
*Did you feel comfortable at your station?*		
Applies	8	72.7
Rather applies	2	18.2
Rather does not apply	1	9.1
Does not apply	0	0
*Did you enjoy working with students at your station?*		
Very much	8	72.7
Much	2	18.2
Partly/partly	1	9.1
Little	0	0
Very little	0	0

The supervisors could rate the location of the GP in the clinic on a scale of 1 (not satisfied) – 10 (very satisfied). Only two supervisors were very satisfied with the location of the GP in the clinic (value 5: *n* = 1, value 7: *n* = 1, value 8: *n* = 3, value 9: *n* = 4). The supervisors could rate the location of their own station on a scale of 1 (not satisfied)–10 (very satisfied). Only three supervisors were very satisfied with the location (value 5: *n* = 1, value 7: *n* = 1, value 8: *n* = 4, value 9: *n* = 2).

The supervisors (*n* = 7, 63.6%) named suggestions for the improvement of the GP (Table 4).

**TABLE 4:** Supervisors' suggestions for the improvement of the first ‘Gero‐Parcours’ set up.

Suggestions for improvement of the ‘Gero‐Parcours’ set up Use more rooms to increase the distance between stations so that there is more silence at each station Better adapt the order of the stations according to the location Combine thematic focuses in one room, for example, sensory organs (seeing, hearing and touching) Organise logical walking routes from one to another station, for example, oral hygiene products (taste and smell), clinical nutrition, assisted oral hygiene Better signage Stopwatches at each station to better time the changes Conscious select the supervisor for the stations with body contact, for example, the wheelchair‐to‐chair transfer station At the assisted oral hygiene station, a mirror at sitting eye level (tiltable mirror) would be helpful At the oral hygiene products (taste and smell) station, a sink for spitting out as part of the neutralisation of tastes would be helpful The station with gerostomatological aids should have even more general aids available in addition to the dental ones A clothes rack for the students' clothes should be placed near the station where the age simulation suits are put on

Supervisors (*n* = 4, 36.4%) did not write in response to the question about suggestions for the GP or had no requests regarding the GP but responded that the GP was good and should be continued as it is. Individual suggestions to improve the GP were: combination with training in long‐term care facilities, videos for an introduction to the station and more theoretical knowledge about some diseases and their treatment at the stations, for example, limitations in seeing.

In this study, 54.5% of the supervisors would always like to supervise the same station in the upcoming GPs. In this study, 27.3% would favour a change for themselves, for example in the next semester, once a daily change within the three course days was named. One supervisor suggested not always supervising the same station in one section but changing between two or three stations in an organised way or going with a group of students from station to station. This would require that the learning objectives of all stations be known to all supervisors.

Five supervisors (45.4%) gave a value of 10 (maximum agreement) on a scale of 0–10 when asked if they personally had gained anything from the course in terms of their assessment of ‘life in old age’ (three supervisors (27.3%) between 1 and 5, three supervisors (27.3%) between 6 and 9).

All supervisors (*n* = 11, 100%) indicated their wish to be back at the next GP. Five supervisors (45.4%) tended to care for the obtained or another station again (no preference for the station), three supervisors (27.3%) wanted to care for the same and three supervisors (27.3%) for another station.

The supervisors rated the students' interest at their specific station and in the GP in general overall mostly as very high or high. (Table 5).

**TABLE 5:** Supervisors' assessment of students' interest in the ‘Gero‐Parcours’ course in 2021 at the three different time points (Day 1–Day 3) the ‘Gero‐Parcours’ took place.

How would you rate the students' interest in the topic at your station?						
	Day 1		Day 2		Day 3	
	*n* = 10		*n* = 9		*n* = 9	
	*n*	%	*n*	%	*n*	%
Very interested	3	27.3	2	22.2	4	44.4
Interested	7	63.6	5	55.6	9	55.6
Less interested	0	0	2	22.2	0	0
Not interested at all	0	0	0	0	0	0

How would you rate the students' interest in the ‘Gero‐Parcours’ in general?						
	Day 1		Day 2		Day 3	
	*n* = 11		*n* = 9		*n* = 9	
	*n*	%	*n*	%	*n*	%
Very interested	8	72.7	5	55.6	5	55.6
Interested	3	27.3	4	44.4	4	44.4
Less interested	0	0	0	0	0	0
Not interested at all	0	0	0	0	0	0

## 4 Discussion

Dentists must increasingly deal with the heterogeneity of their older patients in their daily work.

University teaching is therefore required to prepare dental students not only for diagnosis and treatment but also for dealing with older people with different functional and cognitive capacities and abilities.

For this reason, a very flexible training element with many topics on ageing was developed, the ‘Gero‐Parcours’, which students can complete with many stations and very different topics.

The findings of this study provide insights into the students' experiences and perceptions of the GP, highlighting its positive impact on their understanding of ageing and professional development. Most students felt that the course enhanced their professional knowledge and corrected misconceptions about ageing in patients. Others expressed personal concerns about ageing and the need for respect for older individuals.

When asked to assess the most and least useful GP stations for their future professional lives, students found the station for learning to transfer from a wheelchair to a dental chair to be the most useful. The clinical nutrition station was rated as the least useful. At the end of the survey, students evaluated various aspects of the GP course, and the majority agreed that their expectations for the course were met. Almost 98% of the students would recommend the age awareness simulation training. These findings suggest that practical, hands‐on stations are valued more highly by students and that there may be a need to better integrate the relevance of clinical nutrition into the curriculum, highlighting its importance in geriatric care and potentially adjusting the approach to teaching this aspect to increase its perceived value. This insight could help refine the GP to better address the needs and perceptions of students.

The supervisors generally expressed satisfaction with their assigned stations and the available equipment but reported insufficient time for station preparation. Supervisors provided valuable suggestions for improving the GP setup. About 54.5% of supervisors expressed a preference for supervising the same station in future GPs, while 27.3% favoured changing stations periodically. Furthermore, supervisors felt that the students' interest in the GP and their respective stations remained consistently high across the 3 days of the course. All supervisors wished to participate in future GPs, with 45.4% wanting to care for the same or another station and 27.3% preferring a change of station.

These results emphasise the importance of involving supervisors in the planning process to ensure that their needs and suggestions are taken into account. The supervisor sees each student during a course at the same station and therefore also gets to see the range of each student's understanding of the station topic. This is important for the future development of a station, which can lead to improvements in preparation time, set‐up and the overall orientation of the station. This collaborative approach could enhance the overall effectiveness and sustainability of the GP course.

### 4.1 Study Design

The study describes the evaluation of a teaching unit by dental students. The setup of the study is based on the circumstances and opportunities for teaching at the University of Leipzig, Germany during the study period. This may make it difficult to replicate the study elsewhere, as it is likely that each university or setting will vary due to local circumstances and resources. Ultimately, the authors want to show in which constellation and arrangement it would be possible. However, the concept must be individually adapted and, if necessary, also evaluated.

Here, the study design resulted from the number of groups of two in each cohort. If a cohort has more students, a group of three participants can be formed or the GP can be extended by another station. There are various stations available that have not now been used with the students, but there is experience with the stations from training sessions with dentists (Tables S1–S5).

### 4.2 Study Limitations

The GP was always held at the same time in the dental education 1 week before the students started treating their own patients. It is therefore the time when the students encounter older people, outside their private lives, in a professional situation. Since these first patient‐doctor contacts in the study are unfamiliar, it can certainly be helpful to deal with the possible limitations of patients. Further studies would have to follow to clarify whether preclinical training would also be useful, especially from the point of view of students perhaps going into dental practice early on for clerkships.

With some stations the training is not quite easy, here it would be better if the stations (e.g. simulation loss of information and simulation perceptual disorder) could be supervised by a physiotherapist. The station Clinical nutrition was accompanied by a nutritionist at the University of Leipzig, Germany**,** which is highly recommended.

The extent to which there is a sustained increase in understanding of the ageing process and limitations would need to be addressed in the longer term with a longitudinal study. There is also evidence from a review on physician empathy that empathy could decline over time in medical school and as a resident [19].

The question of age discrimination in dental and medical students is often raised. Dental students in many countries were questioned about this subject using various instruments [20]. The students at all four Swiss universities confirmed that the mandatory subject of senior dentistry is important and is taught sufficiently. In the survey, the Geriatric Attitudes Scale (GAS) questionnaire was used. It showed that the attitude of dental students towards older people is acceptable but could be improved [21]. At the Zurich dental clinic, simulations of age‐related limitations are also used successfully [22]. Interventions, especially those targeting empathy, to improve attitudes of health care students are effective. Improving the attitudes of future dentists could improve the management and quality of dental care for the elderly [23]. The lack of knowledge about the ageing process will reinforce ageist attitudes in health care that may have been acquired at an early age. Therefore, empathy‐based interventions should be addressed in several stations of the GP.

Since the survey of the students regarding the GP was conducted anonymously, it can be assumed that the students provided honest responses. However, a bias towards socially desirable answers cannot be completely ruled out but appears unlikely.

### 4.3 Recommendations

The GP is a very variable teaching element. The students found the GP to be a helpful training tool. It can be easily adapted to the number of students and supervisors as well as to the time available. It is also feasible to divide it into different times in the course. GP should be included as a permanent element in the compulsory dental education curriculum.

There is also the possibility of incorporating other parcours stations, thereby increasing parcours time and personnel involvement. The utilisation of an obesity suit or Heimlich grip simulator [24], among others, could be considered. However, any potential expansion of the course should not have the geriatric patient theme obliterated for students by other topics, such as emergency exercises. It would have to be ensured that the GP is exclusively dedicated to ageing and that there is a separation to possible other training topics.

As a dentist with experience in treating older adults, it appears noteworthy to consider the evaluation of students regarding which stations they find meaningful and which ones they do not. Stations such as Clinical Nutrition were deemed unimportant, despite the fact that aspects such as malnutrition [25] and the cariogenicity of nutrition supplements [26] play a significant role in the daily practice of a geriatric dentist. It is possible that students who are just starting their clinical activities may lack a holistic approach to patient care and therapy at this stage.

In future studies, it should be investigated whether this attitude changes with an increase in dental experience or if it is assessed differently by experienced dentists. Furthermore, from the authors' perspective, it would be advisable to align the content of the GP (Grade Point) with the challenges faced in the everyday work with older adults. Additionally, a change in students' assessment of such rotations could potentially be facilitated by providing a clearer introduction to the topic beforehand.

Additionally, the GP can be implemented not only in the pregraduate training of dentists. It is also possible to integrate the educational instrument into the postgraduate training of dentists and their teams to strengthen the field of senior dentistry and special care dentistry.

The general simulation of old age (such as hearing and visual impairment, gait instability and tremor) is in the foreground and could be used by students of other subjects in the same way. In addition, special subject‐specific focal points can be incorporated into the ‘Gero‐Parcours’. In dentistry, for example, it would be the experience of brushing another person's teeth or, with a visual deficit, having one's teeth brushed by a stranger, such as a nurse. The design in the number of stations, the length of the training units and the number of supervisors must be adapted to local needs.

## 5 Conclusion

The dentistry students at the University of Leipzig, Germany have confirmed that they have gained a better idea of the limitations of ageing and their understanding of older people has increased by completing the GP. This is evidenced by the positive feedback from the students and their willingness to recommend the course. The teaching evaluation data showed an increase in students' awareness and appreciation of the challenges faced by older adults. It is highly likely that GP could help to reduce any age‐discriminating reservations that may exist and increase understanding of the challenge of treating older people. Therefore, based on these findings, the authors recommend the inclusion of the GP as a mandatory component in the dental education curriculum to better prepare students for geriatric patient care.

## Conflicts of Interest

The authors declare no conflicts of interest.

## Supporting information


**Table S1.** Domain 1: Physical limitations stations to be selected in the ‘Gero‐Parcours’.
**Table S2.** Domain 2: Cognitive or sensory impairments stations to be selected in the Gero‐Parcours.
**Table S3.** Domain 3: Equipment and aids stations to be selected in the Gero‐Parcours.
**Table S4.** Domain 4: Handling and management stations to be selected in the Gero‐Parcours.
**Table S5.** Domain 5: Other topics stations to be selected in the Gero‐Parcours.

## Data Availability

The data that support the findings of this study are available on request from the corresponding author. The data are not publicly available due to privacy or ethical restrictions.
