# Supplementary material for: Simulation of Age‐Related Limitations of Patients in Patient‐Centred Dental Education
Source: Eur J Dent Educ. 2025 Feb 17;29(2):356–66. doi: 10.1111/eje.13075 (PMC12006703; doi:10.1111/eje.13075)
Supplement: Supplementary file 1 — Table S1. Domain 1: Physical limitations stations to be selected in the ‘Gero‐Parcours’. Table S2. Domain 2: Cognitive or sensory impairments stations to be selected in the Gero‐Parcours. Table S3. Domain 3: Equipment and aids stations to be selected in the Gero‐Parcours. Table S4. Domain 4: Handling and management stations to be selected in the Gero‐Parcours. Table S5. Domain 5: Other topics stations to be selected in the Gero‐Parcours. [file EJE-29-356-s001.docx]

**Table S1** **Domain 1: Physical limitations** stations to be selected in the in the Gero-Parcours.

| **Stations for physical limitations** | |
| --- | --- |
| **Name of the station** | **Learning objective** |
| Age Simulation Suit  AgeMan ® | - Experience the simulation of physical limitation |
| Simulation of walking insecurity | - Describe own perception - Name characteristics of the altered gait - Name measures that would make the situation more comfortable - Derive and try out recommendations for action for dealing with people with gait disorders in practice |
| Simulation Hemiplegia | - Experience the simulation of physical limitation |
| Simulation Dyspnoea | - Describe own perception - Name negative feelings and find their cause - Name measures that would have made the situation more pleasant - Derive recommendations for action for dealing with people with dyspnoea in the practice (constructional measures, handling, medical aspects, clinical aspects) |
| Simulation Hand tremor | - Experience the simulation of physical limitation |
| Incontinence | - Experience the challenges of incontinence with incontinence pads - Report on accompanying considerations (e.g. shame, fear of overcoming; difficulty putting on pads, etc.). |

**Table S2 Domain 2: Cognitive or sensory impairments** stations to be selected in the in the Gero-Parcours.

| **Stations for sensory and cognitive impairment** | |
| --- | --- |
| **Name of the station** | **Learning objective** |
| Age Simulation Suit  AgeMan ® | - Experience the simulation of cognitive or sensory impairments |
| Simulation  Eye disease | - Experience the simulation of sensory impairments |
| Simulation Hearing impairment | - Reflect on difficulties and facilitating aspects - Getting to know hearing aids with the aid of hearing aid dummies - Learn how to make conversations in the dental office better for people with hearing loss |
| Simulation Dementia | - Describe own perception - Name negative feelings and find their cause - Name measures that would have made the situation more pleasant - Derive recommendations for action for dealing with people with dementia in practice (environment, handling) |
| Simulation Loss of information | - Using the sensory illusion, experience the consequences of disturbed body perception and derive measures for appropriate handling. |
| Simulation Perceptual disorder | - To perceive the body tactile-sensory, senso-motoric and nociceptive by means of therapy mirrors using simple exercises. - Understand that seniors can have subjective perceptions without a direct objective basis and can react accordingly (unpredictably). - Gain understanding of the use of mirror neurons in patient care (e.g., showing mouth opening, brushing teeth in front of the mirror, friendly face) |
| Oral hygiene products  (taste and smell) | - Experience oral hygiene products used by patients |

**Table S3** **Domain 3: Equipment and aids** stations to be selected in the Gero-Parcours.

| **Stations for equipment and aids** | |
| --- | --- |
| **Name of the station** | **Learning objective** |
| Gerostomatological aids | - Experience of taste and smell of aids |
| Nursing care aids | - Get to know nursing aids and their area of usage |
| Transfer aids | - Get to know transfer aids and their area of usage |
| Mobile equipment | - Get to know mobile equipment and their area of usage - Get to know different mobile care systems |

**Table S4** **Domain 4: Handling and management** stations to be selected in the Gero-Parcours.

| **Stations for handling and management** | |
| --- | --- |
| **Name of the station** | **Learning objective** |
| Identifying emotions | - To recognise and name emotions - Learn the difference and relevance of face recognition in seniors - To find reasons why it is important, especially for older people who speak little or not at all, to orientate themselves to emotions as non-verbal communication. |
| Simulation teeth cleaning by others | - Learn and experience how assisted oral hygiene feels for the person whom is getting brushed their teeth |
| Wheelchair transfer | - Experience sitting in a wheelchair yourself - Experience how a transfer from wheelchair to chair takes place and feels. |

**Table S5 Domain 5: Other topics** stations to be selected in the Gero-Parcours.

| **Stations for handling and management** | |
| --- | --- |
| **Name of the station** | **Learning objective** |
| Medication interaction | - Identify drug interactions between the existing drugs in the medication plan of a geriatric patient and a planned therapy (e.g., tooth extraction), local anaesthetics, antibiotics, analgesics and the existing diagnoses. - Plan precautionary measures using a drug reference book |
| Clinical nutrition | - Get to know and experience clinical nutrition products and their application / area of usage |
| Ethical case discussion | - Receive an overview of the thematic introduction to ethical case analysis. |
| Oral Health Care Manager  (OHCM) | - Get to know the concept of the OHCM |
